# Applications of artificial intelligence and machine learning in dynamic pathway engineering

**DOI:** 10.1042/BST20221542

**Published:** 2023-09-01

**Authors:** Charlotte Merzbacher, Diego A. Oyarzún

**Affiliations:** 1 School of Informatics, University of Edinburgh, Edinburgh, U.K.; 2 The Alan Turing Institute, London, U.K.; 3 School of Biological Sciences, University of Edinburgh, Edinburgh, U.K.

**Keywords:** artificial intelligence, machine learning, metabolic engineering, synthetic biology

## Abstract

Dynamic pathway engineering aims to build metabolic production systems embedded with intracellular control mechanisms for improved performance. These control systems enable host cells to self-regulate the temporal activity of a production pathway in response to perturbations, using a combination of biosensors and feedback circuits for controlling expression of heterologous enzymes. Pathway design, however, requires assembling together multiple biological parts into suitable circuit architectures, as well as careful calibration of the function of each component. This results in a large design space that is costly to navigate through experimentation alone. Methods from artificial intelligence (AI) and machine learning are gaining increasing attention as tools to accelerate the design cycle, owing to their ability to identify hidden patterns in data and rapidly screen through large collections of designs. In this review, we discuss recent developments in the application of machine learning methods to the design of dynamic pathways and their components. We cover recent successes and offer perspectives for future developments in the field. The integration of AI into metabolic engineering pipelines offers great opportunities to streamline design and discover control systems for improved production of high-value chemicals.

## Introduction

A key aim in metabolic engineering is the production of high-value chemicals using the metabolic machinery of microorganisms [1,2]. In a typical metabolic engineering pipeline, microbial strains are transformed with enzymatic genes that convert native precursors of the host into target products. However, production is typically limited by multiple factors such as pathway sensitivity to fermentation conditions, accumulation of toxic intermediates, and difficulties in scaling up production. To overcome these challenges, last decade has witnessed the birth of dynamic pathway engineering, a technology where production strains are endowed with built-in feedback control systems. Such control systems can adapt the temporal expression of pathway enzymes in response to changes in cellular or bioreactor conditions [3]. This strategy can improve robustness and diminish the impact of toxic intermediate accumulation, gene expression burden, and other common challenges encountered in applications [4].

Dynamic pathways contain two core components [5]: a backbone production pathway and a set of biosensors that control enzymatic expression in response to metabolite signals. But assembling these systems requires bringing together various disparate molecular components such as catalytic enzymes, metabolite-sensing proteins and genetic elements (e.g. promoters or ribosomal binding sites). The implementation of these systems thus requires costly experimental work for assembling, testing and fine-tuning the system components. Computational methods can help accelerating the design cycle with effective tools for *in silico* modelling and simulation of system performance. To date, such computational tools have been largely dominated by kinetic models using ordinary differential equations. Most recently there has been an increased interest in methods from artificial intelligence (AI) and machine learning [6], owing to their flexibility and ability to detect patterns in complex datasets.

**Figure 1.**
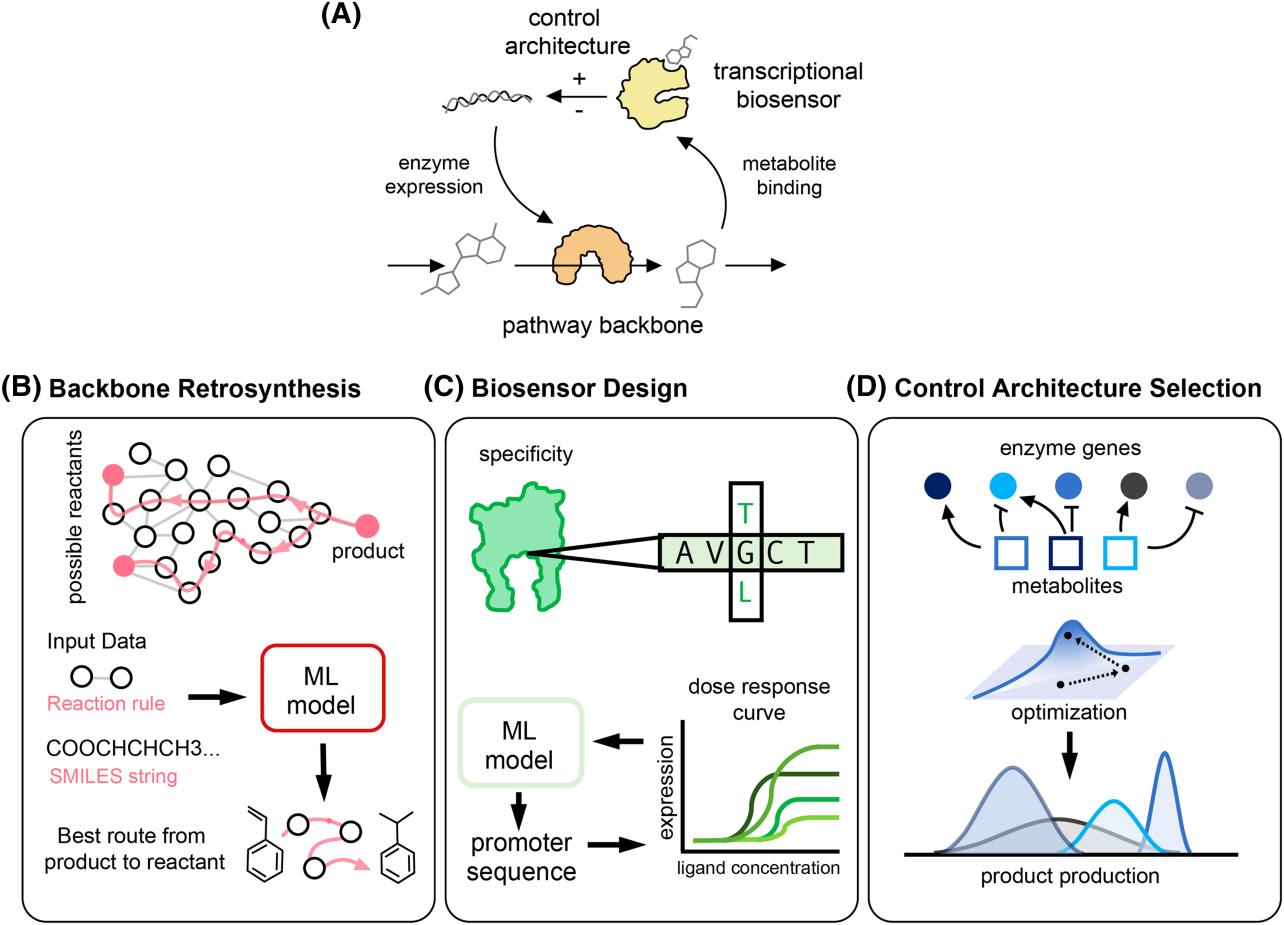
Applications areas of machine learning in dynamic pathway engineering: retrosynthesis, biosensor design, and circuit architecture design. (**A**) Exemplar dynamic pathway whereby metabolites bind to transcriptional biosensors that control the temporal enzyme expression. (**B**) Pathway assembly begins with retrosynthesis of the pathway backbone from native metabolic substrates. Retrosynthesis algorithms predict a given reactant and enzyme which produce the desired product. Machine learning models can be trained on reaction rules or SMILES strings to find the best route from substrates to products [12–15]. (**C**) Metabolite biosensors such as transcription factors or RNA aptamers can be engineered to bind to small molecule ligands [16,17]; progress in protein design guided by machine learning offers exciting routes for the design of ligand-specific biosensors [18,19]. The biosensor dose-response curves can be tuned by changing the promoter sequence or other non-coding genetic elements. Several works have built sequence-to-expression machine learning models that can be employed for the design of such non-coding sequences [20–23]. (**D**) Specific pathway dynamics can be achieved by different control architectures that differ in their implementation costs. The selection of optimal architectures can be aided with optimization methods from machine learning [24–26].

Here, we discuss recent applications of AI and machine learning to aid the design of dynamic pathways. We focus on three aspects of pathway design where machine learning methods have the potential to provide substantial benefits over traditional modelling approaches (Figure 1): pathway assembly via retrosynthesis, design of small molecule biosensors, and the selection of suitable control architectures. For conciseness, we do not discuss details of specific machine learning models, as this is an extensive subject beyond the scope of this review. For a primer on AI and machine learning for biological applications, we refer the reader to the excellent review by Greener et al. [7]. We restrict this review to dynamic pathway engineering, as machine learning applications for static pathways has been covered extensively elsewhere in the literature [6,8–10].

## Pathway retrosynthesis

The first step when designing a production pathway is the identification of enzymatic conversion routes from host metabolites to the target product. Finding such routes involves specifying sequences of reactions steps catalyzed by enzymes that need to expressed in the host of interest. This is a pathway retrosynthesis problem [11] for which numerous computational tools have been developed [27–30]. Typical approaches to retrosynthesis employ template-based strategies, whereby databases of expert-curated pathways and substrate-enzyme pairs are converted into reaction rules. Computational algorithms are then employed to find suitable pathway components and stoichiometries among a combinatorially large design space. These tools produce retrosynthesis networks linking target compounds to metabolites of the host strain, typically ranking the possible pathways based on enzyme availability, performance, product and intermediate toxicities, or theoretical yield.

Machine learning algorithms are finding a growing number of applications in pathway retrosynthesis. For example, retrosynthesis software packages incorporate supervised machine learning models to score candidate pathways based on their ability to retrieve the correct product [28]. Baylon et al. [31] built a machine learning retrosynthesis pipeline with two stages: first, a neural network predicts a group of rules which can be applied to the target chemical, and then a second network predicts a specific chemical transformation within a predicted group of rules. Another approach relied on reinforcement learning to build a tree search algorithm that selects chemical transformations and then ranks the results based on chemical similarity between the current transformation and the native chemical reaction [12]. It has been shown that expert curation can improve the accuracy of machine learning methods, as compared with either of them in isolation [32]. Recent work has also focussed on using graph neural networks (GNNs) for chemical retrosynthesis [33] and their application to biochemical pathways holds substantial promise.

Most recently, progress in large language models has triggered a new wave of template free retrosynthesis algorithms. These work by training machine learning models directly on molecular representations such as SMILES strings and learn chemical reaction rules from a vast corpus of chemical structure data. An initial attempt at the problem was made using an encoder-decoder structure with recurrent neural networks [13]. Following the enormous success of the Transformer architecture [34], several works employed it for pathway retrosynthesis with prediction accuracy surpassing those of template-based methods [14,35,36]. Extensions of this work include architecture modification [37] as well as training on raw patent data rather than SMILES strings, which appears to learn reaction description information in addition to the reaction details [38].

## Design of metabolite biosensors

Biosensors are used throughout metabolic engineering as screening or strain selection tools, and have been built to respond to many signals, including cellular stress responses, temperature, and small molecules [17]. In the case of dynamic pathway engineering, robust production requires up- or down-regulation of enzyme expression in response to metabolic signals. To this end, genetically-encoded metabolite biosensors have been widely adopted to close the loop between pathway activity and enzyme expression. Biosensors employed so far are mostly based on metabolite-responsive transcription factors [5] or RNA aptamers [39], both of which can be used to control gene expression in response to a target metabolite of interest.

Biosensor design comprises primarily two tasks: engineering specificity/affinity toward a target metabolite [17], and engineering the shape of the biosensor dose-response curve, including key parameters as its sensitivity, dynamic range, and leaky expression levels [40]. Modifications to affinity or specificity are typically done with tools from protein or DNA engineering techniques [16]. While not specifically aimed at biosensor design, a large portion of current work at the interface of AI and synthetic biology focuses on protein engineering [41,42]. Significant advances in protein structure prediction algorithms such as AlphaFold2 can learn sequence representations that are predictive of protein secondary and tertiary structure [43,44]. Unsupervised language models have made significant progress in learning high-level protein representations that are predictive of both structure and function [45]. These developments are revolutionizing the predictive design of proteins with novel or improved functions and offer exciting opportunities for biosensor design in dynamic pathway engineering. Beyond protein design, a number of works developed machine learning pipelines to design or improve metabolite-responsive RNA devices. For example, Groher et al. [46] employed supervised learning to improve the function of a tetracycline-dependent riboswitch composed of two aptamers, and other works have incorporated models of RNA secondary structure for the design of S-adenosyl methionine (SAM) riboswitches, one of the most well studied for metabolite-responsive RNA aptamers [47]. A number of other approaches have employed deep learning models of varied complexity for the design of RNA toehold switches that respond to small molecules [20,48,49].

The design of biosensor dose-response curves, on the other hand, has primarily relied on controlling transcriptional and translational efficiency via non-coding elements such as promoters, ribosomal binding sites and terminators [17,50]. Thanks to progress in high-throughput DNA synthesis and sequencing, there is a growing interest in massively parallel reporter assays [51,52] to characterize sequence-function associations [53], and a number of works have employed deep learning to build models for the design of promoters [21,23] and sequences that impact translational efficiency [22,54]. These sequence-to-expression models can be particularly powerful for design, as they can be wrapped into sampling or optimization routines to discover sequences with improved phenotypes [21,55,56]. Using the *lac* repressor as a model system, machine learning algorithms have also been employed to design sequences that influence the shape of the dose-response curve [57]; the work by Zhou et al. [58] applied such approach to improve the dynamic range of a malonyl-CoA responsive transcription factor. Several approaches to response curve engineering have also utilized natural motifs found in related organisms. For example, Ding et al. [59] employed ribosomal binding site data to built a machine learning model that allows predictable tuning of the dynamic range of a glucarate biosensor. Wang et al. [60] successfully used a generalized adversarial networks to generate synthetic promoters after being trained on *Escherichia coli* promoter activity data. Recent work employed GANs to generate entire regulatory sequences with models trained on natural sequences [61].

In many applications of interest, there are few or no biosensors that can respond to intermediates of a specific pathway of interest [62]. To bridge this knowledge gap, several groups have assembled databases of metabolites and transcription factor interactions [63–65]. These datasets can potentially be employed to train machine learning models for biosensor discovery and expand the range of detectable metabolites, particularly considering recent successes in molecular discovery using phenotypic screening data [66,67].

## Design of control architectures

Once a production pathway and the required metabolite biosensors have been established, the next step is the design of a control architecture, i.e. to decide how and which enzymes should be controlled by the biosensor. This is an important design decision because similar control systems can be built with several combinations of positive and negative feedback loops. Such architectures can differ substantially in their complexity and cost of implementation, for example because they require a different number of engineered promoters and transcription factors. To date, the selection of control architectures has been done largely on a trial and-error basis guided by pathway-specific knowledge [5], or with the use of computational pathway models based on differential equations [68]. Several works have employed such models to identify architectures that can support a specific production phenotype [69–72], analyze their temporal dynamics [73–76], and identify architectures that optimize production [77–79].

Recently, several studies have proposed the use of machine learning methods for optimizing the architecture of biological circuits [80,81]. Work by Hiscock [24] exploited gradient descent algorithms commonly employed for training machine learning models to find gene circuit architectures that matches a desired temporal output. Another recent work by Shen et al. [26] employed recurrent neural networks to design synthetic gene circuits, while Frank [82] used automatic differentiation methods from machine learning to select optimal architectures in transcription factor circuits. This body of work has focussed mostly on genetic circuits that do not interact with metabolic pathways. In the case of dynamic pathway engineering, a recent work proposed the use of Bayesian optimization, a technique widely used for model selection in deep learning, to simultaneously optimize control architectures and biosensor dose-response curves [25]. The use of machine learning approaches for circuit design allows exploring large design spaces in a computationally efficient manner, and provides a first step toward integrated design pipelines aimed at dynamic pathway engineering.

## Conclusions

AI and machine learning are rapidly being adopted across many biological design tasks [6,83,84]. In the case of dynamic pathway engineering, recent works highlight how such methods can assist in various stages of the pathway design process. Here, we have discussed such progress along three key directions: pathway retrosynthesis, biosensor design, and control architecture design. The pace and depth of deployment of AI varies significantly across these three areas. For pathway retrosynthesis, the enormous success of language models already has produced new approaches to discover enzymatic conversion routes from host intermediates to target products. In the case of biosensor design, there are numerous AI approaches that support tasks in protein and DNA sequence engineering, which are both required for optimizing biosensor function; while most of these methods have not been specifically tailored for biosensor engineering yet, their increasing adoption will likely permeate to the design of metabolite-responsive molecular mechanisms. Finally, the design of control architectures is the most recent application area of AI in dynamic pathway engineering, and offers exciting avenues for the development of powerful algorithms to screen competing designs and identify those that meet specifications and experimental implementation constraints.

As the current literature shows, machine learning methods have so far been applied to a wide variety of design tasks, many of which require different input data modalities, model architectures and strategies for performance evaluation. Although this flexibility endows designers with a wide range of powerful algorithms, it comes at the cost of large data requirements for model training. Progress in laboratory automation and high-throughput screening are paving the way such data-rich approach for biological design. The development of biofoundries across the globe [85] together with progress in self-driving laboratories [86] offer exciting opportunities for large-scale data acquisition, which can pave the way for the systematic integration of AI and machine learning into pathway design pipelines.

The interface between AI and dynamic pathway engineering is a relatively new and evolving field, with much of the recent work is still at a proof-of-concept stage. Future efforts will likely place an increasing focus on more user-friendly software tools that can bring this technology into the hands of wetlab practitioners, much like in other areas that enjoy a growing number of bespoke software packages [87–89]. One area of particular interest is the use of active learning for pathway design. Active learning is a machine learning paradigm where the model selects the most informative designs to implement, thereby reducing the number of experiments required to explore the design space effectively. Several software packages such as BioAutomata [90], ART [91], ActiveOpt [92], and METIS [93] have implemented active learning pipelines for the design of static production pathways. In the case of dynamic pathways, however there is a pressing lack of comprehensive computational tools that support end-to-end system design. Given the complexity and number of designable components of dynamic pathways, the application of active learning tools could lead to important efficiency gains in implementation and prototyping. With the growing number of applications of machine learning in pathway engineering, and the continued efforts to develop comprehensive software packages, we can expect significant advancements in this area in the coming years that will support the wider adoption of AI and machine learning for strain design.

## Perspectives

- Dynamic pathway engineering offers promising routes for building robust production strains, but these require assembly of many biological components into complex circuits. Computational methods can rapidly screen potential designs *in silico*, thus accelerating the navigation of large and experimentally intractable design spaces.
- There is a growing interest in artificial intelligence methods for the design of dynamic pathways, particularly for pathway retrosynthesis, design of metabolite-responsive biosensors, and the optimization of circuit architectures. Machine learning models can improve over classic algorithms and help solve previously intractable design problems.
- Progress in laboratory automation and high-throughput screening will pave the way for more data-centric approaches to biological design, and enable the wider adoption of AI and machine learning in the field.

## Open Access

Open access for this article was enabled by the participation of University of Edinburgh in an all-inclusive *Read & Publish* agreement with Portland Press and the Biochemical Society under a transformative agreement with JISC.

## Abbreviations

AI: artificial intelligence
GNNs: graph neural networks
SAM: S-adenosyl methionine

## References

[1] Chae, T.U., Choi, S.Y., Kim, J.W., Ko, Y.-S. and Lee, S.Y. (2017) Recent advances in systems metabolic engineering tools and strategies. Curr. Opin. Biotechnol. 47, 67–82 10.1016/j.copbio.2017.06.007 28675826

[2] Stephanopoulos, G.N., Aristidou, A.A. and Nielsen, J. (1998) Metabolic Engineering: Principles and Methodologies, Academic Press 10.1016/B978-0-12-666260-3.X5000-6

[3] Ni, C., Dinh, C.V. and Prather, K.L.J. (2021) Dynamic control of metabolism. Annu. Rev. Chem. Biomol. Eng. 12, 519–541 10.1146/annurev-chembioeng-091720-125738 33784176

[4] Liu, D., Mannan, A.A., Han, Y., Oyarzún, D.A. and Zhang, F. (2018) Dynamic metabolic control: towards precision engineering of metabolism. J. Ind. Microbiol. Biotechnol. 45, 535–543 10.1007/s10295-018-2013-9 29380150

[5] Hartline, C.J., Schmitz, A.C., Han, Y. and Zhang, F. (2021) Dynamic control in metabolic engineering: theories, tools, and applications. Metab. Eng. 63, 126–140 10.1016/j.ymben.2020.08.015 32927059 PMC8015268

[6] Faulon, J.-L. and Faure, L. (2021) *In silico*, *in vitro*, and *in vivo* machine learning in synthetic biology and metabolic engineering. Curr. Opin. Chem. Biol. 65, 85–92 10.1016/j.cbpa.2021.06.002 34280705

[7] Greener, J.G., Kandathil, S.M., Moffat, L. and Jones, D.T. (2022) A guide to machine learning for biologists. Nat. Rev. Mol. Cell Biol. 23, 40–55 10.1038/s41580-021-00407-0 34518686

[8] Kim, G.B., Kim, W.J., Kim, H.U. and Lee, S.Y. (2020) Machine learning applications in systems metabolic engineering. Curr. Opin. Biotechnol. 64, 1–9 10.1016/j.copbio.2019.08.010 31580992

[9] Lawson, C.E., Martí, J.M., Radivojevic, T., Jonnalagadda, S.V.R., Gentz, R., Hillson, N.J. et al. (2021) Machine learning for metabolic engineering: a review. Metab. Eng. 63, 34–60 10.1016/j.ymben.2020.10.005 33221420

[10] Presnell, K.V. and Alper, H.S. (2019) Systems metabolic engineering meets machine learning: a new era for data-driven metabolic engineering. Biotechnol. J. 14, 1800416 10.1002/biot.201800416 30927499

[11] Lin, G.-M., Warden-Rothman, R. and Voigt, C.A. (2019) Retrosynthetic design of metabolic pathways to chemicals not found in nature. Curr. Opin. Syst. Biol. 14, 82–107 10.1016/j.coisb.2019.04.004

[12] Koch, M., Duigou, T. and Faulon, J.-L. (2019) Reinforcement learning for bioret rosynthesis. ACS Synth. Biol. 9, 157–168 10.1021/acssynbio.9b00447 31841626

[13] Liu, B., Ramsundar, B., Kawthekar, P., Shi, J., Gomes, J., Nguyen, Q.L. et al. (2017) Retrosynthetic reaction prediction using neural sequence-to-sequence models. ACS Cent. Sci. 3, 1103–1113 10.1021/acscentsci.7b00303 29104927 PMC5658761

[14] Yang, Q., Sresht, V., Bolgar, P., Hou, X., Klug-McLeod, J.L., Butler, C.R. et al. (2019) Molecular transformer unifies reaction prediction and retrosynthesis across pharma chemical space. Chem. Commun. 55, 12152–12155 10.1039/C9CC05122H 31497831

[15] Yu, T., Boob, A.G., Volk, M.J., Liu, X., Cui, H. and Zhao, H. (2023) Machine learning-enabled retrobiosynthesis of molecules. Nat. Catal. 6, 137–151 10.1038/s41929-022-00909-w

[16] Ding, N., Zhou, S. and Deng, Y. (2021) Transcription-factor-based biosensor engineer ing for applications in synthetic biology. ACS Synth. Biol. 10, 911–922 10.1021/acssynbio.0c00252 33899477

[17] Liu, D., Evans, T. and Zhang, F. (2015) Applications and advances of metabolite biosensors for metabolic engineering. Metab. Eng. 31, 35–43 10.1016/j.ymben.2015.06.008 26142692

[18] Quijano-Rubio, A., Yeh, H.-W., Park, J., Lee, H., Langan, R.A., Boyken, S.E., et al. (2021) *De novo* design of modular and tunable protein biosensors. Nature 591, 482–487 10.1038/s41586-021-03258-z 33503651 PMC8074680

[19] Wu, Z., Kan, S.B.J., Lewis, R.D., Wittmann, B.J. and Arnold, F.H. (2019) Machine learning-assisted directed protein evolution with combinatorial libraries. Proc. Natl Acad. Sci. U.S.A. 116, 8852–8858 10.1073/pnas.1901979116 30979809 PMC6500146

[20] Angenent-Mari, N.M., Garruss, A.S., Soenksen, L.R., Church, G. and Collins, J.J. (2020) A deep learning approach to programmable RNA switches. Nat. Commun. 11, 5057 10.1038/s41467-020-18677-1 33028812 PMC7541447

[21] Kotopka, B.J. and Smolke, C.D. (2020) Model-driven generation of artificial yeast promoters. Nat. Commun. 11, 2113 10.1038/s41467-020-15977-4 32355169 PMC7192914

[22] Nikolados, E.-M., Wongprommoon, A., Aodha, O.M., Cambray, G. and Oyarzún, D.A. (2022) Accuracy and data efficiency in deep learning models of protein expression. Nat. Commun. 13, 7755 10.1038/s41467-022-34902-5 36517468 PMC9751117

[23] Vaishnav, E.D., de Boer, C.G., Molinet, J., Yassour, M., Fan, L., Adiconis, X. et al. (2022) The evolution, evolvability and engineering of gene regulatory DNA. Nature 603, 455–463 10.1038/s41586-022-04506-6 35264797 PMC8934302

[24] Hiscock, T.W. (2019) Adapting machine-learning algorithms to design gene circuits. BMC Bioinformatics 20, 214 10.1186/s12859-019-2788-3 31029103 PMC6487017

[25] Merzbacher, C., Aodha, O.M. and Oyarzún, D.A. (2023) Bayesian optimization for design of multiscale biological circuits. ACS Synth. Biol. 12, 2073–2082 10.1021/acssynbio.3c00120 37339382 PMC10367132

[26] Shen, J., Liu, F., Tu, Y. and Tang, C. (2021) Finding gene network topologies for given biological function with recurrent neural network. Nat. Commun. 12, 3125 10.1038/s41467-021-23420-5 34035278 PMC8149884

[27] Carbonell, P. (2021) Synthetic biology design tools for metabolic engineering. In Microbial Cell Factories Engineering for Production of Biomolecules, (Singh, V., ed.) pp. 65–77, Academic Press, Cambridge, Massachusetts, USA.

[28] Delépine, B., Duigou, T., Carbonell, P. and Faulon, J.-L. (2018) Retropath2. 0: a retrosynthesis workflow for metabolic engineers. Metab. Eng. 45, 158–170 10.1016/j.ymben.2017.12.002 29233745

[29] Finnigan, W., Hepworth, L.J., Flitsch, S.L. and Turner, N.J. (2021) Retrobiocat as a computer-aided synthesis planning tool for biocatalytic reactions and cascades. Nat. Catal. 4, 98–104 10.1038/s41929-020-00556-z 33604511 PMC7116764

[30] Otero-Muras, I. and Carbonell, P. (2021) Automated engineering of synthetic metabolic pathways for efficient biomanufacturing. Metab. Eng. 63, 61–80 10.1016/j.ymben.2020.11.012 33316374

[31] Baylon, J.L., Cilfone, N.A., Gulcher, J.R. and Chittenden, T.W. (2019) Enhancing retrosynthetic reaction prediction with deep learning using multiscale reaction classification. J. Chem. Inf. Model. 59, 673–688 10.1021/acs.jcim.8b00801 30642173

[32] Badowski, T., Gajewska, E.P., Molga, K. and Grzybowski, B.A. (2020) Synergy between expert and machine-learning approaches allows for improved retrosynthetic planning. Angew. Chem. Int. Ed. Engl. 59, 725–730 10.1002/anie.201912083 31750610

[33] Liu, C.H., Korablyov, M., Jastrzębski, S., Włodarczyk-Pruszyński, P., Bengio, Y. and Segler, M. (2022) RetroGNN: fast estimation of synthesizability for virtual screening and *de novo* design by learning from slow retrosynthesis software. J. Chem. Inform. Model. 62, 2293–2300 10.1021/acs.jcim.1c01476 35452226

[34] Vaswani, A., Shazeer, N., Parmar, N., Uszkoreit, J., Jones, L., Gomez, A.N. et al. (2017) Attention is all you need. Adv. Neural Inform. Process. Syst. 30. 10.48550/arXiv.1706.03762

[35] Tetko, I.V., Karpov, P., Van Deursen, R. and Godin, G. (2020) State-of-the-art augmented NLP transformer models for direct and single-step retrosynthesis. Nat. Commun. 11, 5575 10.1038/s41467-020-19266-y 33149154 PMC7643129

[36] Zheng, S., Rao, J., Zhang, Z., Xu, J. and Yang, Y. (2019) Predicting retrosynthetic reactions using self-corrected transformer neural networks. J. Chem. Inform. Model. 60, 47–55 10.1021/acs.jcim.9b00949 31825611

[37] Kim, E., Lee, D., Kwon, Y., Park, M.S. and Choi, Y.-S. (2021) Valid, plausible, and diverse retrosynthesis using tied two-way transformers with latent variables. J. Chem. Inf. Model. 61, 123–133 10.1021/acs.jcim.0c01074 33410697

[38] Kreutter, D., Schwaller, P. and Reymond, J.-L. (2021) Predicting enzymatic re actions with a molecular transformer. Chem. Sci. 12, 8648–8659 10.1039/d1sc02362d 34257863 PMC8246114

[39] Dykstra, P.B., Kaplan, M. and Smolke, C.D. (2022) Engineering synthetic RNA devices for cell control. Nat. Rev. Genet. 23, 215–228 10.1038/s41576-021-00436-7 34983970 PMC9554294

[40] Mannan, A.A., Liu, D., Zhang, F. and Oyarzún, D.A. (2017) Fundamental design principles for transcription-factor-based metabolite biosensors. ACS Synth. Biol. 6, 1851–1859 10.1021/acssynbio.7b00172 28763198

[41] Freschlin, C.R., Fahlberg, S.A. and Romero, P.A. (2022) Machine learning to navigate fitness landscapes for protein engineering. Curr. Opin. Biotechnol. 75, 102713 10.1016/j.copbio.2022.102713 35413604 PMC9177649

[42] Pham, C., Stogios, P.J., Savchenko, A. and Mahadevan, R. (2022) Advances in engineering and optimization of transcription factor-based biosensors for plug-and play small molecule detection. Curr. Opin. Biotechnol. 76, 102753 10.1016/j.copbio.2022.102753 35872379

[43] Baek, M., DiMaio, F., Anishchenko, I., Dauparas, J., Ovchinnikov, S., Lee, G.R., et al. (2021) Accurate prediction of protein structures and interactions using a three-track neural network. Science 373, 871–876 10.1126/science.abj8754 34282049 PMC7612213

[44] Jumper, J., Evans, R., Pritzel, A., Green, T., Figurnov, M., Ronneberger, O., et al. (2021) Highly accurate protein structure prediction with Alphafold. Nature 596, 583–589 10.1038/s41586-021-03819-2 34265844 PMC8371605

[45] Rives, A., Meier, J., Sercu, T., Goyal, S., Lin, Z., Liu, J., et al. (2021) Biological structure and function emerge from scaling unsupervised learning to 250 million protein sequences. Proc. Natl Acad. Sci. U.S.A. 118, e2016239118 10.1073/pnas.2016239118 33876751 PMC8053943

[46] Groher, A.-C., Jager, S., Schneider, C., Groher, F., Hamacher, K. and Suess, B. (2018) Tuning the performance of synthetic riboswitches using machine learning. ACS Synth. Biol. 8, 34–44 10.1021/acssynbio.8b00207 30513199

[47] Fernandez-de Cossio-Diaz, J., Hardouin, P., du Moutier, F.-X.L., Di Gioacchino, A.., Marchand, B., Ponty, Y. et al. (2023) Designing molecular RNA switches with restricted Boltzmann machines. bioRxiv 10.1101/2023.05.10.540155 PMC12714719 41413030

[48] Riley, A.T., Robson, J.M. and Green, A.A. (2023) Generative and predictive neural networks for the design of functional RNA molecules. bioRxiv 10.1101/2023.07.14.549043 PMC12050331 40320400

[49] Valeri, J.A., Collins, K.M., Ramesh, P., Alcantar, M.A., Lepe, B.A., Lu, T.K. et al. (2020) Sequence-to-function deep learning frameworks for engineered riboregulators. Nat. Commun. 11, 5058 10.1038/s41467-020-18676-2 33028819 PMC7541510

[50] Qin, L., Liu, X., Xu, K. and Li, C. (2022) Mining and design of biosensors for engineering microbial cell factory. Curr. Opin. Biotechnol. 75, 102694 10.1016/j.copbio.2022.102694 35158313

[51] Gilliot, P.-A. and Gorochowski, T.E. (2022) Design and analysis of massively parallel reporter assays using forecast. Methods Mol. Biol. 255, 41–56 10.1007/978-1-0716-2617-73 36227538

[52] Nikolados, E.-M. and Oyarzún, D.A. (2023) Deep learning for optimization of protein expression. Curr. Opin. Biotechnol. 81, 102941 10.1016/j.copbio.2023.102941 37087839

[53] Tack, D.S., Tonner, P.D., Pressman, A., Olson, N.D., Levy, S.F., Romantseva, E.F. et al. (2023) Precision engineering of biological function with large-scale measurements and machine learning. PLoS ONE 18, e0283548 10.1371/journal.pone.0283548 36989327 PMC10057847

[54] Höllerer, S., Papaxanthos, L., Gumpinger, A.C., Fischer, K., Beisel, C., Borgwardt, K. et al. (2020) Large-scale DNA-based phenotypic recording and deep learning enable highly accurate sequence-function mapping. Nat. Commun. 11, 3551 10.1038/s41467-020-17222-4 32669542 PMC7363850

[55] Linder, J., Bogard, N., Rosenberg, A.B. and Seelig, G. (2020) A generative neural network for maximizing fitness and diversity of synthetic DNA and protein sequences. Cell Syst. 11, 49–62.e16 10.1016/j.cels.2020.05.007 32711843 PMC8694568

[56] Liu, X., Gupta, S.T.P., Bhimsaria, D., Reed, J.L., Rodríguez-Martínez, J.A., Ansari, A.Z. et al. (2019) *De novo* design of programmable inducible promoters. Nucleic Acids Res. 47, 10452–10463 10.1093/nar/gkz772 31552424 PMC6821364

[57] Tack, D.S., Tonner, P.D., Pressman, A., Olson, N.D., Levy, S.F., Romantseva, E.F. et al. (2021) The genotype-phenotype landscape of an allosteric protein. Mol. Syst. Biol. 17, e10179 10.15252/msb.202010179 33784029 PMC8009258

[58] Zhou, Y., Yuan, Y., Wu, Y., Li, L., Jameel, A., Xing, X.-H. et al. (2022) Encoding genetic circuits with DNA barcodes paves the way for machine learning assisted metabolite biosensor response curve profiling in yeast. ACS Synth. Biol. 11, 977–989 10.1021/acssynbio.1c00595 35089702

[59] Ding, N., Yuan, Z., Zhang, X., Chen, J., Zhou, S. and Deng, Y. (2020) Pro grammable cross-ribosome-binding sites to fine-tune the dynamic range of transcription factor based biosensor. Nucleic Acids Res. 48, 10602–10613 10.1093/nar/gkaa786 32976557 PMC7544201

[60] Wang, Y., Wang, H., Wei, L., Li, S., Liu, L. and Wang, X. (2020) Synthetic promoter design in *Escherichia coli* based on a deep generative network. Nucleic Acids Res. 48, 6403–6412 10.1093/nar/gkaa325 32424410 PMC7337522

[61] Zrimec, J., Fu, X., Muhammad, A.S., Skrekas, C., Jauniskis, V., Speicher, N.K. et al. (2022) Controlling gene expression with deep generative design of regulatory DNA. Nat. Commun. 13, 5099 10.1038/s41467-022-32818-8 36042233 PMC9427793

[62] Koch, M., Pandi, A., Borkowski, O., Batista, A.C. and Faulon, J.-L. (2019) Custom-made transcriptional biosensors for metabolic engineering. Curr. Opin. Biotechnol. 59, 78–84 10.1016/j.copbio.2019.02.016 30921678

[63] d'Oelsnitz, S., Love, J.D., Diaz, D.J. and Ellington, A.D. (2022) Groovdb: a database of ligand-inducible transcription factors. ACS Synth. Biol. 11, 3534–3537 10.1021/acssynbio.2c00382 36178800 PMC13292809

[64] Koch, M., Pandi, A., Delépine, B. and Faulon, J.-L. (2018) A dataset of small molecules triggering transcriptional and translational cellular responses. Data Brief 17, 1374–1378 10.1016/j.dib.2018.02.061 29556520 PMC5854866

[65] Tellechea-Luzardo, J., Lázaro, H.M., López, R.M. and Carbonell, P. (2023) Sensbio: an online server for biosensor design. BMC Bioinformatics 24, 71 10.1186/s12859-023-05201-7 36855083 PMC9972687

[66] Smer-Barreto, V., Quintanilla, A., Elliott, R.J.R., Dawson, J.C., Sun, J., Campa, V.M. et al. (2023) Discovery of senolytics using machine learning. Nat. Commun. 14, 3445 10.1038/s41467-023-39120-1 37301862 PMC10257182

[67] Stokes, J.M., Yang, K., Swanson, K., Jin, W., Cubillos-Ruiz, A., Donghia, N.M. et al. (2020) A deep learning approach to antibiotic discovery. Cell 180, 688–702.e13 10.1016/j.cell.2020.01.021 32084340 PMC8349178

[68] Kim, O.D., Rocha, M. and Maia, P. (2018) A review of dynamic modeling approaches and their application in computational strain optimization for metabolic engineering. Front. Microbiol. 9, 1690 10.3389/fmicb.2018.01690 30108559 PMC6079213

[69] Chaves, M. and Oyarzún, D.A. (2019) Dynamics of complex feedback architectures in metabolic pathways. Automatica 99, 323–332 10.1016/j.automatica.2018.10.046

[70] Dunlop, M.J., Keasling, J.D. and Mukhopadhyay, A. (2010) A model for improving microbial biofuel production using a synthetic feedback loop. Syst. Synth. Biol. 4, 95–104 10.1007/s11693-010-9052-5 20805930 PMC2923299

[71] Oyarzún, D.A. and Chaves, M. (2015) Design of a bistable switch to control cellular uptake. J. R. Soc. Interface 12, 20150618 10.1098/rsif.2015.0618 26674196 PMC4707844

[72] Reznik, E., Kaper, T.J. and Segrè, D. (2013) The dynamics of hybrid metabolic-genetic oscillators. Chaos 23, 013132 10.1063/1.4793573 23556969

[73] Anesiadis, N., Kobayashi, H., Cluett, W.R. and Mahadevan, R. (2013) Analysis and design of a genetic circuit for dynamic metabolic engineering. ACS Synth. Biol. 2, 442–452 10.1021/sb300129j 23654263

[74] Boada, Y., Vignoni, A., Picó, J. and Carbonell, P. (2020) Extended metabolic biosensor design for dynamic pathway regulation of cell factories. iScience 23, 101305 10.1016/j.isci.2020.101305 32629420 PMC7334618

[75] Liu, D. and Zhang, F. (2018) Metabolic feedback circuits provide rapid control of metabolite dynamics. ACS Synth. Biol. 7, 347–356 10.1021/acssynbio.7b00342 29298043

[76] Oyarzún, D.A. and Stan, G.-B.V. (2013) Synthetic gene circuits for metabolic control: design trade-offs and constraints. J. R. Soc. Interface 10, 20120671 10.1098/rsif.2012.0671 23054953 PMC3565798

[77] de Hijas-Liste, G.M., Balsa-Canto, E., Ewald, J., Bartl, M., Li, P., Banga, J.R. et al. (2015) Optimal programs of pathway control: dissecting the influence of pathway topology and feedback inhibition on pathway regulation. BMC Bioinformatics 16, 1–13 10.1186/s12859-015-0587-z 25982966 PMC4433072

[78] Stevens, J.T. and Carothers, J.M. (2015) Designing RNA-based genetic control systems for efficient production from engineered metabolic pathways. ACS Synth. Biol. 4, 107–115 10.1021/sb400201u 25314371

[79] Verma, B.K., Mannan, A.A., Zhang, F. and Oyarzún, D.A. (2021) Trade-offs in biosensor optimization for dynamic pathway engineering. ACS Synth. Biol. 11, 228–240 10.1021/acssynbio.1c00391 34968029

[80] Patra, P., Disha, B.R., Kundu, P., Das, M. and Ghosh, A. (2022) Recent advances in machine learning applications in metabolic engineering. Biotechnol. Adv. 62, 108069 10.1016/j.biotechadv.2022.108069 36442697

[81] Volk, M.J., Lourentzou, I., Mishra, S., Vo, L.T., Zhai, C. and Zhao, H. (2020) Biosystems design by machine learning. ACS Synth. Biol. 9, 1514–1533 10.1021/acssynbio.0c00129 32485108

[82] Frank, S.A. (2022) Optimization of transcription factor genetic circuits. Biology 11, 1294 10.3390/biology11091294 36138773 PMC9495410

[83] Carbonell, P., Radivojevic, T. and García Martín, H. (2019) Opportunities at the inter section of synthetic biology, machine learning, and automation. ACS Synth. Biol. 8, 1474–1477 10.1021/acssynbio.8b00540 31319671

[84] Sieow, B.F., De Sotto, R., Seet, Z.R.D., Hwang, I.Y. and Chang, M.W. (2023) Synthetic biology meets machine learning. Methods Mol. Biol. 2553, 21–39 10.1007/978-1-0716-2617-7 36227537

[85] Hillson, N., Caddick, M., Cai, Y., Carrasco, J.A., Chang, M.W., Curach, N.C. et al. (2019) Building a global alliance of biofoundries. Nat. Commun. 10, 2040 10.1038/s41467-019-10079-2 31068573 PMC6506534

[86] Martin, H.G., Radivojevic, T., Zucker, J., Bouchard, K., Sustarich, J., Peisert, S. et al. (2023) Perspectives for self-driving labs in synthetic biology. Curr. Opin. Biotechnol. 79, 102881 10.1016/j.copbio.2022.102881 36603501

[87] Chen, K.M., Cofer, E.M., Zhou, J. and Troyanskaya, O.G. (2019) Selene: a pytorch based deep learning library for sequence data. Nat. Methods 16, 315–318 10.1038/s41592-019-0360-8 30923381 PMC7148117

[88] Hérisson, J., Duigou, T., du Lac, M., Bazi-Kabbaj, K., Azad, M.S., Buldum, G. et al. (2022) The automated Galaxy-SynBioCAD pipeline for synthetic biology design and engineering. Nat. Commun. 13, 5082 10.1038/s41467-022-32661-x 36038542 PMC9424320

[89] Nielsen, A.A.K., Der, B.S., Shin, J., Vaidyanathan, P., Paralanov, V., Strychalski, E.A. et al. (2016) Genetic circuit design automation. Science 352, aac7341 10.1126/science.aac7341 27034378

[90] HamediRad, M., Chao, R., Weisberg, S., Lian, J., Sinha, S. and Zhao, H. (2019) Towards a fully automated algorithm driven platform for biosystems design. Nat. Commun. 10, 5150 10.1038/s41467-019-13189-z 31723141 PMC6853954

[91] Radivojević, T., Costello, Z., Workman, K. and García Martín, H. (2020) A machine learning automated recommendation tool for synthetic biology. Nat. Commun. 11, 4879 10.1038/s41467-020-18008-4 32978379 PMC7519645

[92] Kumar, P., Adamczyk, P.A., Zhang, X., Andrade, R.B., Romero, P.A., Ramanathan, P. et al. (2021) Active and machine learning based approaches to rapidly enhance microbial chemical production. Metab. Eng. 67, 216–226 10.1016/j.ymben.2021.06.009 34229079

[93] Pandi, A., Diehl, C., Kharrazi, A.Y., Scholz, S.A., Bobkova, E., Faure, L. et al. (2022) A versatile active learning workflow for optimization of genetic and metabolic networks. Nat. Commun. 13, 3876 10.1038/s41467-022-31245-z 35790733 PMC9256728

